# Serum IL-1β can be a biomarker in children with severe persistent allergic rhinitis

**DOI:** 10.1186/s13223-019-0368-8

**Published:** 2019-09-18

**Authors:** Myung Woul Han, Song Hee Kim, Inbo Oh, Yang Ho Kim, Jiho Lee

**Affiliations:** 1 Department of Otolaryngology, Ulsan University Hospital, University of Ulsan College of Medicine, 877 Bangeojinsunhwan-doro, Dong-gu, Ulsan, 44033 Republic of Korea; 2 Environmental Health Center, University of Ulsan College of Medicine, Ulsan, Republic of Korea; 3 Department of Occupational and Environmental Medicine, Ulsan University Hospital, University of Ulsan College of Medicine, 877 Bangeojinsunhwan-doro, Dong-gu, Ulsan, 44033 Republic of Korea

**Keywords:** Allergic rhinitis, Asthma, Atopy, IL-1β, Biomarker

## Abstract

**Background:**

Allergic rhinitis (AR) is one of the most common diseases globally and usually persists throughout life. In the present study, we aimed to determine whether the expression of inflammatory biomarkers has a relationship with the severity of allergic rhinitis and with comorbid asthma or other allergic diseases in children.

**Methods:**

For diagnosis of AR, the skin prick test was performed to measure the responses to 18 allergens. Blood levels of eosinophils and immunoglobulin E (IgE) were examined. We classified the patients into 2 groups based on the severity of the condition as Group 1 [intermittent AR (IAR) or mild persistent AR (PAR)] and Group 2 (moderate to severe PAR). To determine the expression of inflammatory biomarkers, in serum and several biomarkers (caspase-1, IL-1β, CCL-11, CCL-24 and IL-33) were measured in the serum using enzyme-linked immunosorbent assay (ELISA). Additionally, we analyzed the correlation between clinical variables and the expression of biomarkers (eosinophils count, IL-1β and CCL-24) and the severity of AR.

**Results:**

We found that eosinophils count, IL-1β, a marker of activation of inflammasomes, and CCL-24 were significantly increased in the moderate to severe PAR group (*p* = 0.008, *p* = 0.003, *p* = 0.039). Additionally, the expressions of eosinophil count, IL-1β and CCL-24 were significantly higher in patients with active asthmatic symptoms than in those without these conditions. On univariate analysis, allergic rhinitis in sibling, paternal allergic rhinitis, high expression of eosinophils count, IL-1β and CCL-24, history of active asthma and atopy correlated with severity of AR. Multivariate analysis showed only paternal allergic rhinitis and high expression of IL-1β as significant risk factors of moderate to severe PAR with 6.4 fold and 4.7 fold-increase in risk, respectively (*p* = 0.011 and *p* = 0.030).

**Conclusion:**

In conclusion, this study provides the first evidence that an excessive release of biologically active IL-1β may promote inflammation in severe PAR. It demonstrates that IL-1β can be a biomarker for active allergic diseases such as AR, asthma, and atopy. Moreover, this finding suggests that IL-1B should be investigated as a therapeutic target in severe PAR and other allergic diseases.

## Introduction

Allergic rhinitis (AR) is one of the most common diseases worldwide and usually persists throughout life. The prevalence of self-reported AR is estimated to be approximately 2% to 25% in children. AR is frequently associated with asthma. Compared to other medical conditions, AR might not appear to be serious because it is not associated with severe morbidity or mortality. However, AR reduces the quality of life of many patients by impairing sleep quality and cognitive function and causing irritability and fatigue. It is associated with decreased school and work performance, especially during the peak pollen season [1–3]. In addition, uncontrolled moderate-to-severe AR affects asthma control [4, 5] and AR itself can be a risk factor for uncontrolled asthma in children [6]. According to the allergic rhinitis and its impact on asthma (ARIA) classification, AR is subdivided into intermittent allergic rhinitis (IAR) and persistent allergic rhinitis (PAR). The severity of allergic rhinitis can be classified as mild or moderate-severe depending on the severity of the symptoms and their impact on social life, and school and work performance [3, 7].

The signs and symptoms of AR are the result of an IgE-mediated allergic reaction involving different cells, mediators, cytokines, chemokines, neuropeptides, and other components of a complex immunological network [8]. Various biomarkers can be obtained by several more or less non-invasive sampling methods to evaluate the nasal allergic response and the disease activity in AR [8–10]. While there are a lot of studies about diagnostic biomarkers that help identify patients with allergic rhinitis, limited studies have been conducted on prognostic biomarkers that are of value in evaluating the risk of disease progression or severity of allergic rhinitis [8, 11–14]. Assessment of inflammation or prediction of severity using biomarkers offers a more objective and direct read-out that can contribute to our understanding of the mechanisms of allergic rhinitis, helps monitor disease severity, and is useful in evaluating the effects of (novel) treatments [8]. A large number of studies investigating various biomarkers for AR have been published over the past decades regarding their diagnostic and/or predictive value [8, 15–17].

Inflammatory biomarkers can aid in establishing the diagnosis, in staging and monitoring of the disease activity/progression, or in predicting or monitoring of a treatment response. Especially in (young) children, reliable, non-invasive biomarkers would be valuable. Cytokine levels and other mediators have been investigated in patients with rhinitis using the nasal fluid [8, 9, 18], either to get more insight regarding the pathogenic condition, for diagnostic purposes, or to measure therapeutic effects. However, no single or specific biomarker for AR has been identified to date, especially in children’s serum, for predicting the severity of the condition [9, 18, 19].

Inflammasomes are key inflammatory signaling platforms that detect microbial substances, sterile environmental insults, and molecules derived from host cells. Activation of inflammasomes promotes caspase-1-mediated secretion of proinflammatory cytokines such as interleukin (IL)-1β and IL-18 and pyroptosis. It is likely that excessive inflammasome activation and IL-1β responses contribute to the complex inflammatory processes in the development of COPD and neutrophilic asthma as well as infection-associated exacerbations that promote progression to more severe disease [20, 21]. IL-1β is a key molecule and its release plays a crucial role in the pathogenesis of allergic asthma [22]. Thus, antagonizing the function of IL-1β holds promise as a potential therapeutic option for the treatment of asthma [20–23].

In the present study, we aimed to determine whether the expression of inflammatory biomarkers has a relationship with the severity of allergic rhinitis and with comorbid asthma or other allergic diseases in children. Moreover, we also tried to analyze the impact of environmental factors on PAR.

## Patients and methods

### Study subjects

Subjects included 3097 children enrolled in the Elementary School Students Cohort (2015–2016) for identifying environmental factors of allergic disease in the Environmental Health Center of Ulsan University Hospital (Ulsan, Korea). The study participants were recruited from 4 elementary schools in Ulsan. Parental informed consent was obtained before enrollment. This study was approved by the institutional ethics review committee of Ulsan University Hospital (approval number 2009-09-061-011). At the time of enrollment, the subjects were given a detailed questionnaire and laboratory tests were performed. All the students responded to the questionnaire and underwent routine medical checkups, including a skin prick test and a blood test for total IgE levels and eosinophil counts. The questionnaires used in this study consisted of the International Society of Asthma and Allergy of Children (ISAAC) survey questions [24], and questions regarding the patients’ socioeconomic status and hazardous environmental factors. The parents or legal guardians of all participants provided written informed consent. We assembled a cohort of patients diagnosed with AR to undertake a nested case–control study. We identified all children who were diagnosed as having allergic rhinitis based on clinical symptoms and objective tests of IgE-mediated skin prick test.

### Diagnosis of allergic rhinitis

We used the clinical definition of AR for diagnosis, namely, symptoms of allergic rhinitis including rhinorrhea, nasal obstruction, nasal itching, and sneezing, which are reversible spontaneously or with treatment, and objective tests of IgE-mediated allergy, such as the skin prick test [2]. As part of the study, the skin prick test was performed during a routine medical examination, to measure the responses to 18 allergens, which were compared to a positive and a negative control. Positive reactions for each allergen were defined as a wheal diameter at least 3 mm greater than that of the negative control.

### Measurement and analysis of serum cytokine, chemokine, and inflammasome levels

Blood levels of eosinophils and immunoglobulin E (IgE) were examined. To determine the expression of inflammatory biomarkers in patients with AR, several biomarkers were measured in the serum. The expression of interleukin (IL)-33 [25] (an epithelial biomarker that plays an important role in the development of allergic diseases), C–C motif chemokine ligand (CCL)-11, CCL-24 [26, 27] (chemokines that induce eosinophils in allergic diseases), and caspase-1 and IL-1β (which represent the activation of inflammasomes) [20, 21] were measured by enzyme-linked immunosorbent assay (ELISA). The details of each of the ELISA kits used in the study are as follows: Human CCL24/Eotaxin-2 ELISA (catalog no. DCC240B), CCL11/Eotaxin ELISA (catalog No. DTX00), IL-33 ELISA (catalog No. D3300), and caspase-1/ICE-1β/IL-1F2 (catalog No. DLB50). The kits were obtained from R & D Systems (Minneapolis, MN).

### Statistical analysis

The independent *t* test and the χ2 test were used to analyze baseline differences. Univariate and multivariate analyses were performed to determine possible relationships between the variables and the development of severe AR. Multiple logistic regression analyses were performed using variables found to be significant (*P *< 0.05) in the univariate analysis and those reported to be associated with the development of severe AR. A *P* value less than 0.05 was considered statistically significant in all analyses. Analyses of the receiver operating characteristic (ROC) curve and the differences in the area under curves (AUC) were used to estimate the diagnostic accuracy of each test.

## Results

### Patient characteristics

We selected 116 children who had experienced AR in the 1 year and in whom the severity of symptoms and duration could be classified according to the ARIA classification. Table 1 presents the characteristics of study participants. The mean age was 8.66 years (range, 6–12 years) and boys and girls were 69 and 47, respectively.

**Table 1:** Characteristics of children with allergic rhinitis (N = 116)

Demographic factors	
Age	8.66 ± 1.67 years (range, 6–12 years)
Boys/girls	69/47
Height (cm)	134.05 ± 11.20
Weight (kg)	31.58 ± 8.58

Laboratory factors	
Eosinophil (%)	4.55 ± 2.54
Eosinophils (count/μl)	316.74 ± 187.72
Total IgE (IU/ml)	441.65 ± 929.42
Caspase (pg/ml)	97.70 ± 46.33
IL-1β (pg/ml)	12.45 ± 13.29
CCL-11 (pg/ml)	84.69 ± 30.35
CCL-24 (pg/ml)	482.53 ± 301.81
IL-33 (pg/ml)	0.75 ± 4.15

The mean eosinophil (%), eosinophils count and IgE levels were 4.55 ± 2.54%, 316.41 ± 187.72 count/μl and 441.65 ± 929.42 IU/ml, respectively. And the mean caspase-1, IL-1β, CCL-11, CCL-24, and IL-33 levels were 97.70 ± 46.33 pg/ml, 12.45 ± 13.29 pg/ml, 30.34 ± 30.35 pg/ml, 482.53 ± 301.81 pg/ml, and 0.75 ± 4.15 pg/ml, respectively.

We classified the patients into 2 groups based on the duration of symptoms and severity of the condition as follows: Group 1 (intermittent allergic rhinitis, IAR and mild persistent allergic rhinitis, PAR) and Group 2 (moderate to severe persistent allergic rhinitis, PAR). There were 79 patients in Group 1 and 37 patients in Group 2.

### Comparison of inflammatory biomarkers

**Fig. 1:**
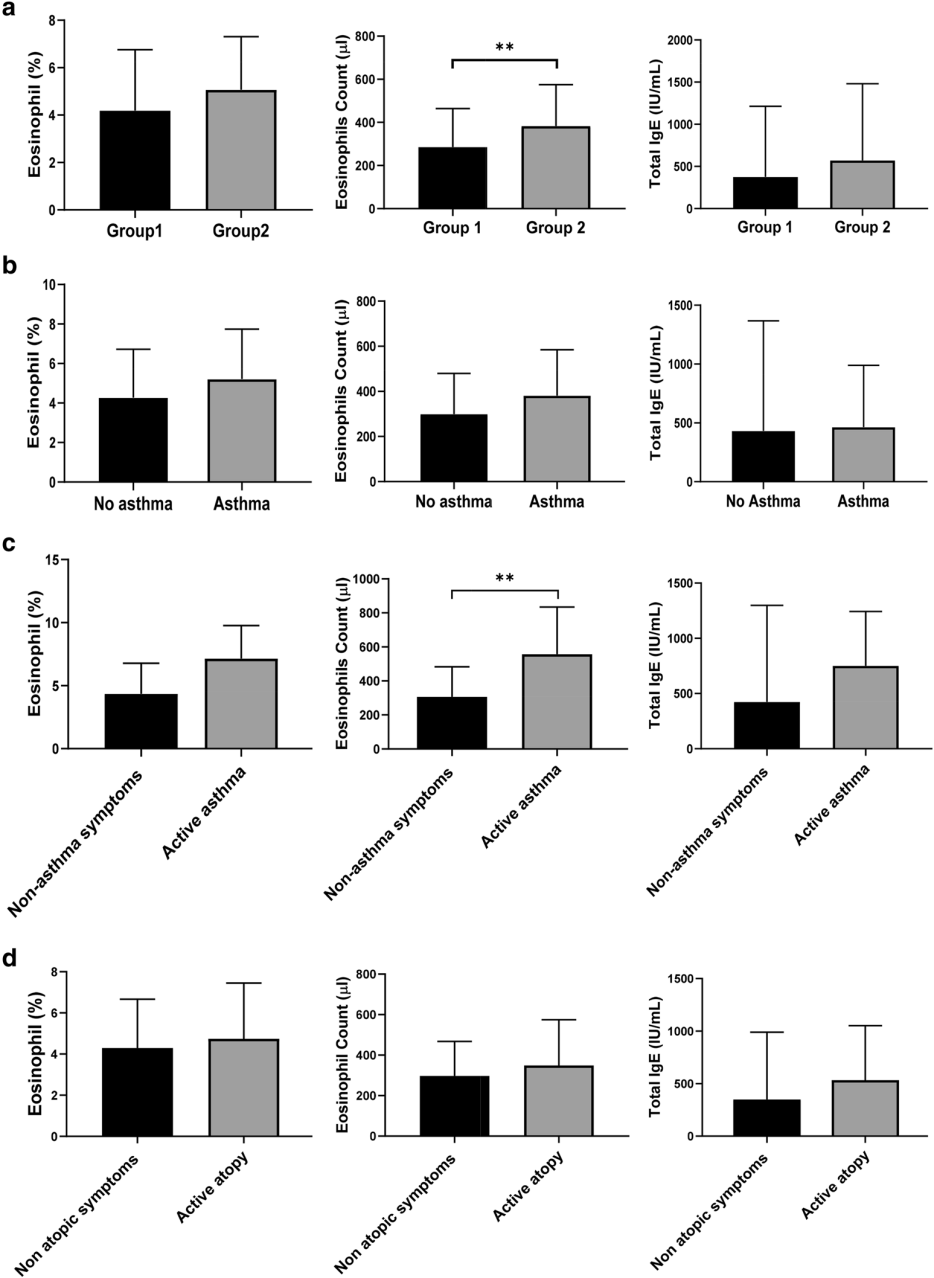
Comparison of the mean concentrations of eosinophil (%), eosinophil count and total IgE of blood in the IAR or mild PAR group (Group 1) and the moderate to severe PAR group (Group 2). According to severity of rhinitis (**a**), asthma history (**b**), active symptom of asthma (**c**), active symptom of atopy (**d**). *P < 0.05. **P < 0.01, *** P < 0.001

At first, we analyzed the eosinophil and IgE level, markers of allergy in each group (Table 2 and Fig. 1). When we compared the levels of the markers, we found that eosinophils count in group 2 was significantly higher than group 1 (*p* = 0.008). But, total IgE level was no significant difference between two groups. And eosinophils count was significantly higher in AR with active asthma than AR without active symptoms (305.9 ± 176.9 vs 557.1 ± 277.3, *p* = 0.003) but, there was no significant difference when it comes to asthma history and atopy (Fig. 1).

**Table 2:** The comparison of blood eosinophil and total IgE level according to severity of allergic rhinitis

	Group 1	Group 2	P value
Eosinophil (%)	4.19 ± 2.56	5.073 ± 2.23	0.075
Eosinophils (count/μl)	285.61 ± 178.72	383.34 ± 191.65	0.008
Total IgE (IU/ml)	376.52 ± 837.01	571.25 ± 909.92	0.258

**Fig. 2:**
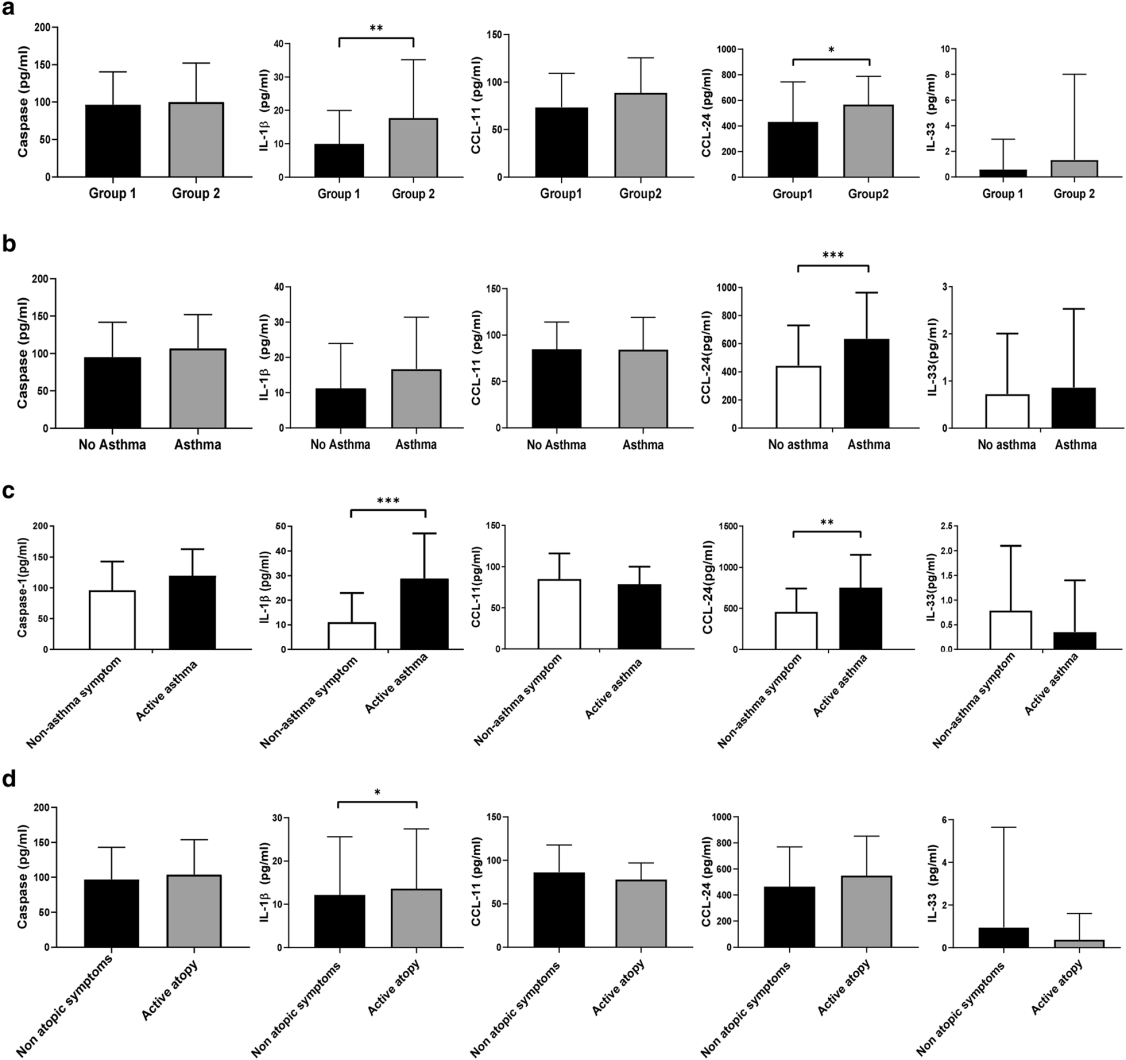
Comparison of the mean concentrations of caspase-1, IL-1β, CCL-11, CCL-24, and IL-33 **a** in the IAR or mild PAR group (Group 1) and the moderate to severe PAR group (Group 2), **b** in children with and without asthma, **c** in children with and without active symptoms of asthma within 1 year and **d** in children with and without active symptoms of atopy (skin rash) within 1 year. *P < 0.05. **P < 0.01, ***P < 0.001. CCL: C–C motif chemokine ligand; IL: interleukin

Next, we investigated the expression of other inflammatory markers, and in group 1, the mean caspase-1, IL-1β, CCL-11, CCL-24, and IL-33 levels were 96.59, 9.98, 82.84, 443.04, and 0.50, respectively. In group 2, the mean caspase-1, IL-1β, CCL-11, CCL-24, and IL-33 levels were 100.08, 17.72, 88.63, 566.83, and 1.32, respectively. When we compared the levels of the inflammatory biomarkers, we found that IL-1β, a marker of activation of inflammasomes, and CCL-24, a marker of activation of eosinophils, were significantly increased in Group 2 *(p* = 0.003, *p* = 0.039, Fig. 2a).

Additionally, we investigated the difference in expression of biomarkers between patients with AR and asthma (N = 23) and those with AR alone (N = 93). In terms of differences in the expression of biomarkers between patients with AR and asthma and patients with AR alone, we found that in the group with asthma, expression of CCL-24 were significantly higher than in the group without asthma (*p* = 0.006, Fig. 2b). And the expression of IL-1β and CCL-24 in patients with activation of asthma (N = 9) in 1 year was significantly higher than in children without activation of asthma (*p* < 0.001 and *p* = 0.004, Fig. 2c). In children with active atopic symptoms (skin rash) within 1 year (N = 37), the expression of IL-1β was significantly higher than in children without atopic symptoms (*p* = 0.049, Fig. 2d). In Group 2, there was no significant difference of eosinophils count, IL-1β and CCL-24 when the expression was compared between patients with AR and asthma (N = 10) and patients with AR alone (N = 27, data was not shown). Collectively, these results suggest that eosinophils count, IL-1β and CCL-24 were overexpressed in children with moderate to severe PAR and with other active allergic diseases and therefore, can be used as a biomarker of exacerbation or activation of allergic diseases including AR. Additionally, we investigated correlation between eosinophil count and IL-1β and CCL-24 expression which overexpression was shown in severe AR and the significant correlation was shown between IL-1β and CCL-24 *(p* = 0.0042), but there was no significant correlation between eosinophils count and IL-1β or eosinophils count and CCL-24 (*p* = 0.8123, *p* = 0.180).

### Independent risk factors for the development of moderate to severe PAR

We also tried to determine whether eosinophils count, IL-1β and CCL-24 expression could be used diagnostically in patients with moderate to severe PAR with classification according to the cut-off value of IL-1β and CCL-24 using ROC curves. We analyzed the correlation between clinical variables including perinatal factors, environmental factors, parental and socioeconomic factors, family history of allergic disease, and activation of other allergic symptoms and the severity of AR. For eosinophils count, with a cut-off value of 285.2 count/μl, there were 79 children with low expression and 37 children with high expression using ROC. For IL-1β, with a cut-off value of 7.98 pg/ml, there were 64 children with low expression and 32 children with high expression using ROC. For CCL-24, with a cut-off value of 471.01 pg/ml, there were 56 children with low expression and 60 children with high expression using ROC. Table 3 presents the results of the univariate analysis of factors with regard to the presence of moderate to severe PAR. On univariate analysis, history of AR in sibling, history of paternal AR, high eosinophils count (≥ 285.2), high expression of IL-1β (≥ 7.98 pg/ml), CCL-24 (≥ 471.01), history of active asthma symptoms within 1 year and active atopic symptoms within 1 year, were associated with the development of moderate to severe PAR (*p* = 0.039, *p* = 0.002, *p* = 0.029, *p* = 0.002, *p* = 0.017, *p* = 0.003, and *p* = 0.050, respectively). Perinatal factors, environmental factors, and socioeconomic factors did not show correlation with the development of moderate and severe PAR. After multivariate analysis, only paternal allergic rhinitis (6.4-fold increase in risk) and high expression of IL-1β (4.7-fold increase in risk), emerged as significant risk factors of moderate to severe PAR (*p* = 0.011 and *p* = 0.030, respectively, Table 4).

**Table 3:** Univariate analysis to determine factors related to the severity of allergic rhinitis

AR severity	Group 1 (N = 79)	Group 2 (N = 37)	*p* value
Perinatal factors			
Delivery			
Normal delivery	46	25	0.415
Delivery by cesarean section	33	12	
Birth weight			
LBW (< 2.5 kg)	9	3	0.749
NBW ≥ 2.5 kg	70	34	
Prematurity			
Prematurity (< 37 weeks)	8	3	0.726
Full-term (≥ 37 weeks)	71	34	
Oxygen therapy in the first week			
No	72	32	0.517
Yes	7	5	
Breastfeeding			
No	17	9	0.812
Yes	62	28	
General anesthesia before 1 year			
No	78	36	0.538
Yes	7	1	
Environmental factors			
Tobacco smoking			
No	62	32	0.293
Yes	16	4	
Distance to major roadway or factory			
< 100 m	53	24	0.835
≥ 100 m	26	13	
Exposure to traffic exhaust			
No	52	24	0.920
Yes	27	13	
Family history of allergy			
Allergic disease in sibling			
No	64	22	0.331
Yes	8	5	
Allergic rhinitis in sibling			
No	50	14	0.039
Yes	25	17	
Paternal allergic disease			
No	60	21	0.176
Yes	15	10	
Paternal allergic rhinitis			
No	55	15	0.002
Yes	23	22	
Maternal allergic disease			
No	62	26	0.778
Yes	14	5	
Maternal allergic rhinitis			
No	48	16	0.074
Yes	29	20	
Activation of other allergic symptoms (history in 1 year)			
Active asthma			
No	76	31	0.029
Yes	3	6	
Active atopic symptoms			
No	62	17	0.002
Yes	18	19	
Active allergic conjunctivitis			
No	57	24	0.516
Yes	22	13	
History of asthma			
No	66	27	0.215
Yes	13	10	
Eosinophils (count/μl)			
Low expression (< 285.2)	45	34	0.017
High expression (≥ 285.2)	12	25	
IL-1β (pg/ml)			
Low expression (< 7.98)	51	13	0.003
High expression (≥ 7.98)	28	24	
CCL-24 (pg/ml)			
Low expression (< 471.01)	43	13	0.050
High expression (≥ 471.01)	36	24	

Group 1: Children with intermittent allergic rhinitis or mild persistent allergic rhinitis; Group 2: children with moderate to severe persistent allergic rhinitis

*AR* Allergic rhinitis, *CCL* C–C motif chemokine ligand,* IL* interleukin,* LBW* low birth weight,* NBW* normal birth weight

**Table 4:** Multivariate analysis to determine factors related to the severity of the persistent allergic rhinitis

	OR	P value	95% CI	
Allergic rhinitis in sibling	1.098	0.295	0.151	1.774
Paternal allergic rhinitis	6.426	0.011	0.086	0.731
Eosinophil count (< 285.2 vs ≥ 285.2)	2.833	0.092	0.143	1.159
IL-1β (< 7.98 vs ≥ 7.98)	4.697	0.030	0.122	0.900
CCL-24 (< 471.01 vs ≥ 471.01)	1.233	0.267	0.205	5.376
Active atopy	2.028	0.154	0.093	1.366
Active asthma	4.070	0.253	0.367	45.138

*CCL* C–C motif chemokine ligand,* CI* confidence interval,* IL* interleukin,* OR* odds ratio

## Discussion

Inflammatory biomarkers can aid in establishing the diagnosis, in staging and monitoring of the disease activity/progression, or in predicting or monitoring of a treatment response. Especially in (young) children, reliable, non-invasive biomarkers would be valuable. Cytokine levels and other mediators have been investigated in patients with rhinitis using the nasal fluid [8, 9, 18], either to get more insight regarding the pathogenic condition, for diagnostic purposes, or to measure therapeutic effects. However, no single or specific biomarker for AR has been identified to date, especially in children’s serum, for predicting the severity of the condition [9, 18, 19]. In children, measurements of inflammatory markers are inconsistent across the different (sampling) techniques reflecting disease heterogeneity, methodological limitations, or varying sensitivity of the biomarker detection techniques [9, 12, 18, 19]. Hence, at this stage, biomarkers cannot be generally recommended as reliable tools to evaluate or treat a child with AR. The research and development of biomarkers using relatively non-invasive techniques, such as using blood and urine samples, will help predict the severity and prognosis in AR and asthma patients and will contribute to the development of new therapeutic targets.

There are no data at present regarding the role of IL-1β in AR in children. Our nested case–control study demonstrated that elevated IL-1β levels and paternal allergic rhinitis have a significant effect on the severity of AR. To the best of our knowledge, this is the first study to compile and evaluate the expression of inflammasome-related proteins in children with AR using a serum sample. The current study had an adequate sample size and used strict inclusion criteria to ensure that a well-characterized group of subjects with moderate to severe PAR (according to ARIA guidelines) could be observed alongside a group of mild PAR subjects. Basically, we investigated the blood IgE and eosinophil level, the marker of allergic response. It is known that nasal eosinophils and basophils result in the late-phase allergic response. Nasal smear eosinophil counts is highly specific criterion for the diagnosis of AR but are poor indicators of the degree, duration, or type of upper or associated lower airway inflammation due to allergy [28]. High blood eosinophil count is a risk factor for incident asthma and eosinophilic inflammation of the airways characterises disease severity in subsets of individuals with severe asthma and there is a direct relationship between eosinophil count and the frequency of asthma exacerbation [29, 30]. It was unknown about the role of blood eosinophils count in AR and we demonstrated that the higher eosinophils count can be biomarker of severity or activation of allergic disease although it was not significant risk factor for severe PAR on multivariate analysis.

In the present study, we also investigated the chemokine ligand levels, that is, CCL11 (also known as eotaxin), and CCL24 (also known as eotaxin-2 or myeloid progenitor inhibitor factor-2), which are all epithelial cell–derived chemokines involved in the pathogenesis of asthma and rhinitis. It is known that CCL5, CCL11, and CCL24 may contribute to the airway recruitment of fibrocytes in severe asthma [26]. CCL24 is likely involved in the pathogenesis of chronic nasal hypereosinophilia; nasal fluid CCL24 levels show a significant correlation with the degree of eosinophilia and clinical symptoms [31, 32]. In our study, CCL-24 was significantly overexpressed in severe PAR and in children with asthma, although it did not show up as a significant risk factor for severe PAR on multivariate analysis. These results suggest that CCL-24 may contribute to airway inflammation in severe allergic rhinitis and active symptomatic asthma. We found the correlation between IL-1β and CC-24 and these biomarkers may play important role in development of inflammation in AR.

The current study demonstrated that IL-1β was strong risk factor to develop of moderate to severe PAR and other active allergic diseases, and therefore, it can be a biomarker of exacerbation or activation of allergic diseases, including AR. The primary role of the inflammasomes is to direct inflammation by controlling unregulated IL-1β release; therefore, IL-1β can be a significant marker of activation of allergic disease or severity. Recent literature has indicated that IL-1β levels are increased in bronchoalveolar lavage fluid and sputum of asthmatic patients compared with those seen in healthy volunteers, and IL-1 blockade can be a novel target for asthma therapy. IL-1ra is a natural anti-inflammatory cytokine that competes with IL-1β for binding to the IL-1 receptor, preventing IL-1β from binding to the IL-1 type I receptor and effectively reduced airway inflammation [33]. Therefore, new therapeutic approaches for the treatment of allergic diseases, such as asthma and severe rhinitis, could be aided by the development of agents that target the IL-1β.

In this study, paternal but not maternal allergic rhinitis had a significant effect on the severity of AR. We cannot guess the reason and meanings accurately, but there is chance that results from higher portion of boys. Recent article indicates a sex dependent association of parental allergic conditions with childhood allergies; maternal allergy increasing the risk in girls and paternal allergy in boys [34]. There were some limitations to our study. First, this study had geographic selection bias, Ulsan of Korea and may not be generalized to all children or adults and second, we only determined expression of inflammatory biomarkers at once.

To date, no single or specific biomarker for allergy, especially for severe PAR, has been identified [9, 18, 35]. As allergy is not one disease, but a collection of a number of allergic conditions, it is therefore not very plausible that one marker would fit all, and therefore, a more holistic approach using a combination of clinical history, clinical readouts, and diagnostic markers will be needed for correct diagnosis.

## Conclusion

In conclusion, this study provides the first evidence for the hypothesis that an excessive release of biologically active IL-1β may promote inflammation in severe PAR. It also demonstrates that IL-1β can be a biomarker of active allergic diseases such as AR, asthma, and atopy. Moreover, this finding suggests that IL-1B should be investigated as a therapeutic target in severe PAR and other allergic diseases. Further studies will help fully elucidate the potential role of IL-1β in AR treatment. Moreover, identification of accurate biomarkers in patients who are unlikely to respond to conventional therapy may promote the development of rational drug combinations that will overcome this problem.

## Data Availability

The datasets used and/or analyzed during the current study are available from the corresponding author on reasonable request.

## Abbreviations

IAR: intermittent allergic rhinitis
PAR: persistent allergic rhinitis
OR: odds ratios
CI: confidence intervals
CCL: C–C motif chemokine ligand
IL: interleukin
LBW: low birth weight
NBW: normal birth weight
